# Case Report: First case of West Nile virus meningoencephalitis in Southwest Michigan in a patient on ixekizumab and prednisone

**DOI:** 10.3389/fmed.2026.1744404

**Published:** 2026-02-09

**Authors:** Henry Zou, Sara Elzalabany, Ingrid Kenyon, Matthew Kelly

**Affiliations:** Homer Stryker M.D. School of Medicine, Western Michigan University, Kalamazoo, MI, United States

**Keywords:** ixekizumab, meningoencephalitis, neuroinvasive disease, prednisolone, West Nile virus

## Abstract

**Background:**

Meningoencephalitis involves inflammation of the meninges and brain parenchyma and is commonly associated with bacterial or viral infection. West Nile virus (WNV) is a mosquito-borne, single-stranded RNA arbovirus that rarely induces neuroinvasive disease. We present the first case of West Nile meningoencephalitis in Southwest Michigan.

**Case report:**

A 58-years-old male with cardiovascular disease, chronic obstructive pulmonary disease, diabetes, chronic kidney disease and psoriasis presented with chest pain, dyspnea, and confusion. Brain imaging was negative, but he developed worsening weakness, nausea, vomiting, fever, and confusion. He received mosquito and tick bites 2 weeks prior. He was started on empiric antibiotic and antiviral therapy and subsequently developed a diffuse morbilliform rash. Initial infectious workup and lumbar puncture were negative, and he was transitioned to solely supportive care. He was discharged after 11 inpatient days following symptomatic improvement, and 6 days later his cerebrospinal fluid was positive for West Nile virus.

**Significance:**

West Nile virus is the most common source of mosquito-borne disease in the mainland USA but <1% present as neuroinvasive disease. Supportive care is the mainstay of treatment, though multiple therapies are under investigation. Our patient’s immunosuppressing medications and multiple comorbidities placed him at greater risk of developing West Nile meningoencephalitis.

## Introduction

Meningoencephalitis refers to inflammation of both the meninges and brain parenchyma most commonly caused by viral and bacterial infection (1). It can present with classic meningitis (fever, headache, neck stiffness, photophobia) and encephalitis (altered mentation, focal neurologic deficits, seizures) symptoms (1). Rash sometimes occurs and is frequently concerning for systemic infection (2). Multiple mosquito- and tick-borne pathogens have also been implicated in meningoencephalitis, including West Nile virus (WNV) (1). WNV is a mosquito-borne, single-stranded RNA arbovirus and represents the leading cause of mosquito-borne disease in the contiguous United States (3). WNV is endemic throughout Michigan, with the highest rates of infection occurring from July to September; however, WNV meningoencephalitis is poorly documented in Michigan (4). Approximately 20% of WNV infections induce symptomatic fever, and under 1% progress to neuroinvasive disease (3). Risk factors for progressing to WNV-induced neuroinvasive disease include immunosuppression, diabetes, hypertension, chronic obstructive pulmonary disease (COPD), and chronic kidney disease (CKD). We present the first case of West Nile meningoencephalitis in Southwest Michigan in a patient with multiple comorbidities taking ixekizumab and prednisone.

## Case presentation

A 58-years-old male with a past medical history of sick sinus syndrome treated with permanent pacemaker placement, chronic obstructive pulmonary disease (COPD), type 2 diabetes, psoriasis on ixekizumab, psoriatic arthritis on long-term prednisone, stage 3 chronic kidney disease (CKD), and coronary artery disease with coronary stenting 1 week prior presented to the hospital for chest pain, shortness of breath, and confusion. Computed tomography (CT) and magnetic resonance imaging (MRI) of the brain showed no acute process; Cardiology was consulted and suspected symptomatic dehydration. He had a mild acute-on-chronic kidney injury which resolved with intravenous fluid resuscitation and was discharged after 3 days of hospitalization. Three days after discharge, the patient returned to the emergency department (ED) with worsening confusion, lightheadedness, bilateral lower extremity weakness, nausea, vomiting, and a fever of 38.9 °C. The patient’s wife revealed that he received a tick bite and multiple mosquito bites 2 weeks prior but denied any recent travel abroad. ED labs showed leukocytosis of 12.4 K/μL with neutrophilic predominance of 9.4 K/μL and mild hypercreatininemia of 1.4 mg/dL. Urinalysis was positive for 3 + proteins and 1 + hemoglobin, but negative for leukocyte esterase, nitrites, and bacteria.

**FIGURE 1:**
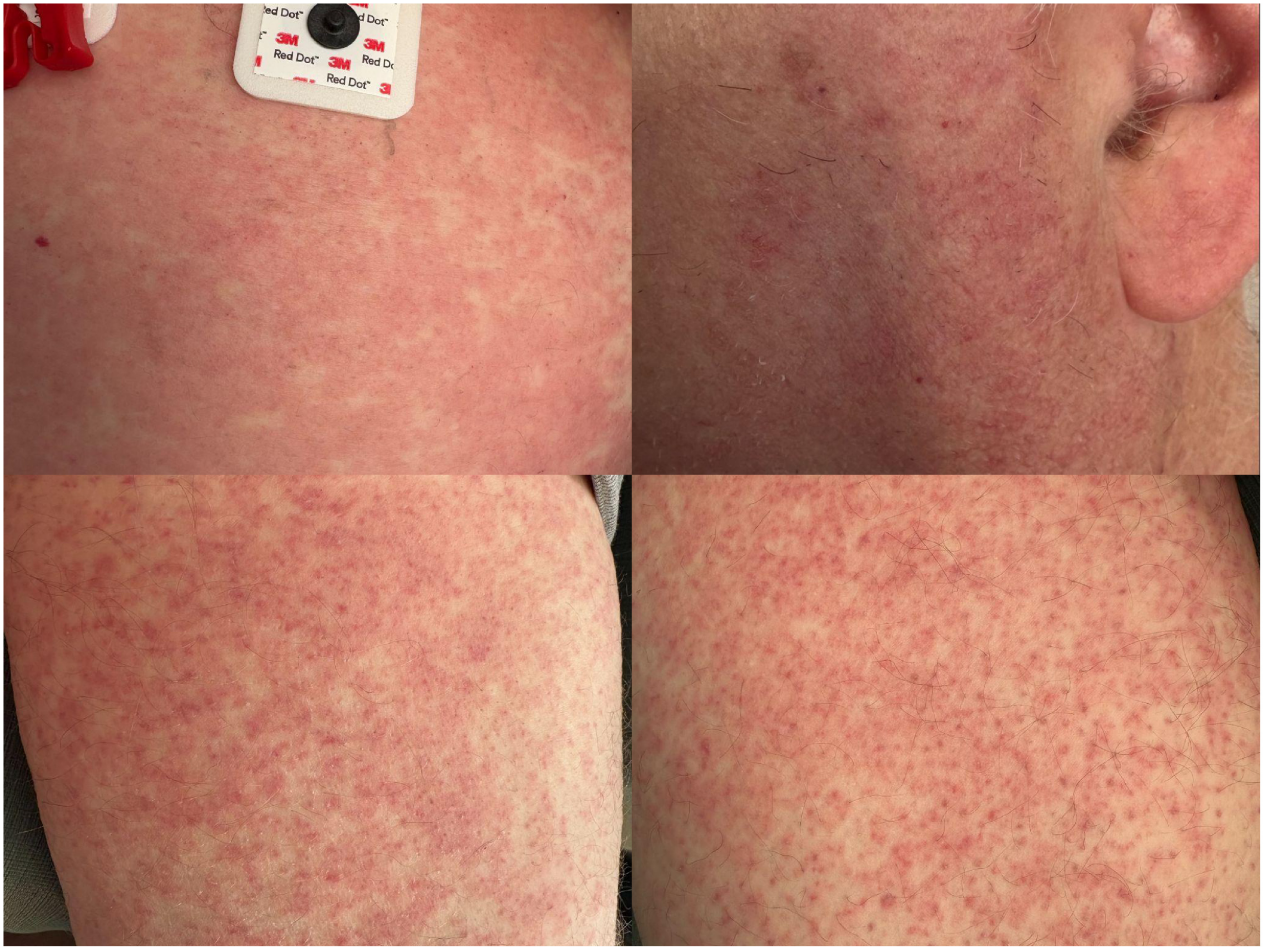
Diffuse morbilliform rash on the chest (top left), face (top right), and bilateral thighs (bottom left and right).

He was admitted inpatient and started on intravenous (IV) empiric treatment for community-acquired meningitis, including ceftriaxone 2 g every 12 h, vancomycin 1.25 g every 18 h, ampicillin 2 g every 4 h to cover bacterial meningitis, and acyclovir 750 mg every 8 h to cover herpes simplex encephalitis. The following morning he developed neck stiffness, recurrent vomiting, and leukocytosis of 12.4 cells/μL. Over the next 2 days he developed hallucinations and headaches with waxing and waning mentation. On hospitalization day three he developed a diffuse, pruritic, and morbilliform rash on the chest, arms, thighs, and face (Figure 1).

Infectious workup including viral nasopharyngeal testing (influenza/COVID-19/respiratory syncytial virus), Lyme and Epstein Barr Virus serologies, and cerebrospinal Venereal Disease Research Laboratory testing were all negative. Repeat brain CT remained unremarkable, and a lumbar puncture showed inflammatory changes (proteinemia at 115 mg/dL, pleocytosis of 25 cells/uL with neutrophilia and mild lymphocytosis) with normal glucose and opening pressure. Blood and cerebrospinal fluid (CSF) bacterial cultures were negative. Two punch biopsies collected from lateral thighs bilaterally showed superficial dermal perivascular chronic inflammatory infiltrate containing rare eosinophils and focal erythrocyte extravasation. These findings indicated dermal hypersensitivity reactions, capillaritis, or pigmented purpuric dermatosis.

Jamestown Canyon virus, Eastern Equine Encephalitis virus, and WNV serologies along with *Anaplasma*, *Ehrlichia*, and Rickettsia polymerase chain reaction (PCR) tests were ordered from the patient’s blood samples and processed at Mayo Clinic laboratories. Chemiluminescent immunoassays were used to detect potential immunoglobulins G (IgG) and M (IgM) generated due to the above diseases. Results were qualitative as they were listed as either positive or negative, but a positive result required exceeding the reference range of detected antibodies in units/mL. The reference range for WNV IgG was ≤1.29 units/mL with a definitively positive result being ≥1.5 units/mL, and the reference range for WNV IgM was ≤0.89 units/mL with a definitively positive result being ≥1.11 units/mL.

Empiric IV antibiotic and antiviral therapies were discontinued after 4 days due to low concern for bacterial etiology or herpes simplex virus encephalitis, and the patient was managed with supportive care including oral doxepin and cetirizine for pruritus with IV fluid resuscitation as needed. Neurologic symptoms including altered mental status, neck pain, hallucinations, and headache began resolving around hospital day seven, but the rash continued to spread until about hospital day nine, at which point it began to subside. He was discharged on hospital day 11 with a 2-weeks course of oral doxycycline 100 mg twice daily due to suspicion for rickettsial disease, oral doxepin for itching, and outpatient primary care follow-up.

Six days after discharge, the patient’s blood tested positive for WNV IgG and IgM but negative for all other pathogens. These results were reported to the local health authorities, who informed the primary team that he was the first documented WNV meningoencephalitis case in Southwest Michigan. He was instructed to stop doxycycline and continue adequate rest and hydration. When he was seen in outpatient follow-up 4 days after discharge, the rash had largely resolved and he was no longer experiencing weakness, fatigue or fevers, though still reported intermittent mild headaches. He was ambulating with a walker and referred to physical therapy by his primary care provider.

## Discussion

West Nile virus is endemic throughout the contiguous USA and Puerto Rico; an annual average of 1298 cases of West Nile-associated neuroinvasive disease (WNNV) were reported from 2014 to 2023 (3). Rare documented complications of WNNV include chorioretinal scarring in a 63-years-old female following inpatient empiric meningoencephalitis antimicrobial therapy (5) and opsoclonus in a 55-years-old female successfully treated with clonazepam and dexamethasone (6).

Supportive care remains the mainstay of WNNV treatment, and while high-titered intravenous WNV-specific immunoglobulin showed promise in animal studies, it did not demonstrate superior efficacy relative to placebo in human randomized controlled trials (7). Investigational therapies include small-molecule cilnidipine, mycophenolate mofetil, nitazoxanide, and teriflunomide as well as two dihydroorotate dehydrogenase inhibitors, but none are recommended by current guidelines (8). Our patient initially underwent empiric antibiotic and antiviral therapy, but ultimately achieved neurologic recovery without severe long-term sequelae with supportive care and symptomatic management.

Immunocompromised status, male sex, diabetes, hypertension, COPD, and CKD all constitute risk factors for WNNV (3, 9). Our male patient has type 2 diabetes, COPD, and stage 3 CKD; furthermore, he is immunocompromised given his long-term use of ixekizumab and prednisone. Ixekizumab is an interleukin-17A inhibitor that can impair antibacterial and antifungal immune defenses, and has been associated with upper respiratory infections and mucocutaneous candidiasis (10). While ixekizumab is not considered a generalized immunosuppressant, emerging evidence indicates that ixekizumab may increase viral susceptibility, with patients older than 65 years found to be at increased risk of contracting herpes zoster (10, 11). While ixekizumab may have increased our patient’s susceptibility to viral infection, there is limited research exploring the increased risk of contracting WNNV in patients younger than age 65.

Prednisone is a generalized immunosuppressant that reduces cytokine production and T-cell function, allowing for higher and prolonged viremia that increases the risk central nervous system invasion and WNNV (12). Although our patient was on a low dose of prednisone (5 mg daily), his long-term use of multiple immunosuppressants and multiple comorbidities likely increased his risk of developing WNNV.

Limitations to our study included the sample size of one, which hinders our clinical generalizability, and the significant delays in obtaining results for our patient’s serologies and PCR tests given their need to be transported and processed at external facilities. This led to our patient being on a prolonged and unnecessary course of antibiotics even after hospital discharge, which represents a major need to optimize our efficiency of definitive diagnosis and treatment for future patients.

In conclusion, we present the first documented case of West Nile meningoencephalitis in Southwest Michigan. Multiple comorbidities and immunosuppressing medications put our patient at greater risk of WNNV, and we encourage clinicians to consider WNNV as a potential differential diagnosis.

## Data Availability

The original contributions presented in this study are included in this article/supplementary material, further inquiries can be directed to the corresponding author.
